# Immunogenicity and Protective Effect of a Virus-Like Particle Containing the SAG1 Antigen of *Toxoplasma gondii* as a Potential Vaccine Candidate for Toxoplasmosis

**DOI:** 10.3390/biomedicines8040091

**Published:** 2020-04-18

**Authors:** Won Hyung Choi, Ji Sun Park

**Affiliations:** 1 Marine Bio Research & Education Center, Kunsan National University, 558 Daehak-ro, Gunsan-si, Jeollabuk-do 54150, Korea; 2 Department of Chemistry, College of Natural Science, Kunsan National University, 558 Daehak-ro, Gunsan-si, Jeollabuk-do 54150, Korea; 1810003@kunsan.ac.kr

**Keywords:** toxoplasmosis, immunogenicity, vaccine, prevention, antigen delivery system

## Abstract

This study was carried out to evaluate the vaccination effect of a virus-like particle (VLP) including the surface antigen 1 (SAG1) of *Toxoplasma gondii* as a potential vaccine for toxoplasmosis. The SAG1 virus-like particles (SAG1-VLPs) were expressed by Sf9 cells, and their expression was confirmed through cloning, RT-PCR analysis, and western blot method. The immunogenicity and vaccine efficacy of SAG1-VLPs were assessed by the antibody response, cytokine analysis, neutralization activity, splenocyte assay, and survival rates through a mouse model. In particular, IgG, IgG1, IgG2a, and IgA were markedly increased after immunization, and the survival rates of *T. gondii* were strongly inhibited by the immunized sera. Furthermore, the immunization of SAG1-VLPs effectively decreased the production of specific cytokines, such as IL-1β, IL-6, TNF-α, and IFN-γ, after parasite infection. In particular, the immunized group showed strong activity and viability compared with the non-immunized infection group, and their survival rate was 75%. These results demonstrate that SAG1-VLP not only has the immunogenicity to block *T. gondii* infection by effectively inducing the generation of specific antibodies against *T. gondii*, but is also an effective antigen delivery system for preventing toxoplasmosis. This study indicates that SAG1-VLP can be effectively utilized as a promising vaccine candidate for preventing or inhibiting *T. gondii* infection.

## 1. Introduction 

Until recently, infectious diseases such as dengue fever, yellow fever, influenza, malaria, middle east respiratory syndrome (MERS), Zika fever, and Ebola have consistently occurred in various countries in the world, which has been causing a serious global threat to many countries and humans. Moreover, a new coronavirus has recently emerged in Wuhan city of China, which has the potential to spread as a serious pandemic worldwide through contact and interaction between humans. Zoonosis induces very serious diseases in human society. From this perspective, *Toxoplasma gondii* is a parasite that is found in all warm-blooded animals, including birds and marine animals. In addition, *T. gondii* is a zoonotic parasite that induces toxoplasmosis in humans by consuming particularly raw or undercooked meat, and by being infected via physical contact with feces of pets and companion animals such as cats. It causes serious chronic diseases through its infection at all ages, including both adults and young children. Biologically, *T. gondii* not only has similar intracellular organelles, such as eukaryotic cells, but also includes various and unique micro-organelles, such as rhoptries, conoids, micronemes, apicoplasts, endosome compartments, and a basal complex. Furthermore, *T. gondii* has an inner membrane complex (IMC) and plasma membrane that consist of a unique double membrane structure, which acts as a protective wall for the survival, cell division, and proliferation of *T. gondii* [1,2,3,4]. In particular, *T. gondii* proliferates through a unique form of cell division, such as amoeba, slipper-animalcule, or bacteria, which indicates a characteristic of endodyogeny. *T. gondii* proliferates and grows into a vacuole membrane by forming a parasitophorous vacuole membrane (PVM) in host cells after infection as a key feature [5,6,7]. 

Recently, zoonotic diseases have been causing a major public health concern worldwide by frequently occurring in various countries, which is a major concern at global, regional, and country levels. Efforts to overcome these challenges of zoonotic diseases have been attempted in various research groups and pharmaceutical industry fields, including global non-profit organizations. In particular, it was reported that extracts derived from medicinal plants and compounds significantly induced anti-*T. gondii* effects/activity in the in vivo and in vitro stages [8,9,10,11,12,13]. Nevertheless, effective drugs of next generation for inhibiting *T. gondii* have not yet been developed. In this regard, the difficulty of developing drugs against parasitic zoonosis not only consistently causes a public health crisis worldwide, but also increases the risk of unknown zoonosis. Although various compounds, biomedicine, and/or vaccines have continuously been developed for treating infectious disease such as Zika fever, malaria, tuberculosis, acquired immune deficiency syndrome (AIDS), influenza, and/or parasite infection in the past decades, people are still widely exposed to various threats, including drug-resistance [14,15,16,17,18,19,20,21,22]. Moreover, viruses and parasites have advanced their biological evolution, as well as evasive techniques, for survival by effectively sustaining the enduring interactions with humans and/or the natural environment. The influenza virus is a major pathogen that consistently causes acute infectious respiratory diseases in humans, and has recently induced a serious public health concern worldwide by overcoming the limits of the species barrier. In addition, the influenza virus consists of various constitutive factors, including seven or eight single-strand RNAs, neuraminidase, hemagglutinin, virus matrix protein M1, proton channel M2, and a lipid bilayer, and its genome has increased the resistance and/or evasion strategy against existing drugs and environmental changes through mutations. For these reasons, the various antigens of pathogens have been utilized as a major factor in solutions for the prevention and treatment of infectious diseases and zoonosis. Furthermore, vaccine techniques and methods through unique antigens (such as DNA, RNA, and proteins) of pathogens have recently advanced, and have been effectively utilized for blocking and preventing infectious diseases [23,24,25,26,27]. However, despite these efforts, an effective vaccine against toxoplasmosis has not yet been developed or launched clinically.

In particular, among the subcellular organelles and unique substances of *T. gondii*, the surface antigen 1 (SAG1) of *T. gondii* plays a key role in mediating cell adhesion to the host cell, which acts as a critical factor when *T. gondii* invades host cells. Furthermore, the SAG1 has not yet been reported as a virus-like particle (VLP) form for the vaccination of *T. gondii* and toxoplasmosis. The studies for developing and/or discovering effective and novel vaccine candidates against *T. gondii* are urgently required. In these aspects, this study started from the hypothesis that SAG1-VLP conflated with SAG1 and the influenza virus matrix protein may induce the protective effect and vaccine efficacy against *T. gondii* infection. For this reason, this study was performed to evaluate the vaccination effect of SAG1-VLP formed by the influenza A matrix protein and the specific SAG1 antigen of *T. gondii* as an effective strategy for preventing and/or blocking toxoplasmosis and *T. gondii* infection, and to confirm the potential and the utility of SAG1-VLP as a vaccine candidate against *T. gondii* infection.

## 2. Materials and Methods

### 2.1. Materials 

Fetal bovine serum (FBS), phosphate buffered saline (PBS), a Bac-to-Bac expression system, serum-free SF900 III medium, and Cellfectin II were purchased from Invitrogen Corporation (Carlsbad, CA, USA). The restriction enzymes were purchased from New England Biolabs Inc. (Ipswich, MA, USA). All other chemicals and reagents were purchased from Sigma-Aldrich Chemical Co., Ltd. (St. Louis, MO, USA), Merck Chemical Co., Ltd. (Darmstadt, Germany), and Donginbio Co., Ltd. (Seoul, Korea). 

### 2.2. Animals 

Balb/c mice (5–6 weeks/female, *n* = 300) were purchased from DBL Co., Ltd. (Chungcheongbuk-do, Korea), and all animals were kept at 23 ± 0.5 °C and in a 12 h-light/dark cycle in a controlled environment of a central animal care facility. Food and water were provided ad libitum to all animals daily during the experiment. All experimental animals and the facility used for this study were maintained and approved (code: KC-2018-0317-02) in accordance with the guidelines of the animal research ethics of the national institutes of health for the care and use of laboratory animals.

### 2.3. Preparation of Parasites and Cell Culture 

*Toxoplasma gondii* RH was purchased from the American Type Culture Collection (Manassas, VA, USA). *T. gondii* RH was maintained through the serial intraperitoneal passage in Balb/C mice. *T. gondii* RH was suspended with 1× PBS, which was injected into the abdominal cavity of Balb/c mice. Five days after the injection, *T. gondii* was collected from the peritoneal fluid of mice kept in the abdominal cavities of the mice before use. *Spodoptera frugiperda* Sf9 cells were cultured in serum-free SF900 III medium (Thermo Fisher Scientific, Gibco^®^, Waltham, MA, USA) at 27 °C in spinner flasks at 80–100 rpm. 

### 2.4. Toxoplasma Gondii Antigen 

*T. gondii* RH was collected from the peritoneal cavity of the mice 5 days after infection. The exudate was separated by centrifugation at 1500 rpm for 5 min at 4 °C, and the cellular debris was carefully removed. The parasites in the supernatant were precipitated by centrifugation at 3000 rpm for 10 min and washed in 1X PBS (phosphate buffered saline). The *T. gondii* lysate antigen (TLA) was prepared through the sonication of 10 cycle/min for 10 min with 1 min intervals. The concentration of antigen was quantified by an Eppendorf BioSpectrometer (Eppendorf, Seoul, Korea), and the samples were stored at −80 °C until use.

### 2.5. The Expression of T. gondii-Specific Antigen and Influenza A virus Matrix 

Total RNA was isolated from the *T. gondii* RH strain using the RNeasy Mini Kit (Qiagen, Hilden, Germany) and obtained from the groups following the manufacturer’s recommended procedure, which was quantified in an Eppendorf BioSpectrometer (Eppendorf, Seoul, Korea). One pair of primers of *T. gondii* SAG1 and the influenza A virus matrix protein was designed according to the sequence of the selected specific gene in NCBI GenBank as follows: *T. gondii* SAG1 (accession number: JX045360, 1011 bp) and influenza A virus segment 7 matrix protein (accession number: EF467824, 1027 bp). The specific gene of *T. gondii* was synthesized into complementary DNA (cDNA) through the one-step RT-PCR kit (Bioneer, Daejeon, Korea) with primers as follows: SAG1 (accession number: JX045360, Forward: 5-GAATTCATGTCGGTTTCGCTGCACCAC-3 and Reverse: 5-AGGCCTTCACGCGACACAAGCTGCGAT-3; *Eco*RI and *Stu*I sites are underlined). The influenza matrix gene was synthesized from Bioneer (Daejeon, Korea), and the influenza matrix gene for the cloning step was amplified by the PCR kit with primers, as follows: Influenza matrix (accession number: EF467824, Forward: 5-GGATCCAGCGAAAGCAGGTAGATATTG-3 and Reverse: 5- GCGCGCAGGTAGTTTTTTACTCCAGCT-3; *Bam*HI and *Bss*HII sites are underlined). The specific genes (SAG1 and matrix protein 1) were cloned using the pFastBac vector system (Invitrogen, Carlsbad, CA, USA) and the restriction enzymes (New England Biolabs, MA, USA), and analyzed by electrophoresis in a 1.2% (*w*/*v*) agarose gel containing 10 μg/mL ethidium bromide at 120 V for 1 h, which was visualized under ultraviolet illumination.

### 2.6. The Production and Preparation of Recombinant Baculovirus (rBV) 

The rBV expressing the specific genes of *T. gondii* and influenza matrix protein 1 was generated through a Bac-to-Bac expression system according to the manufacturer’s instructions as follows: Briefly, the transformation of these genes was performed using a DH10 Bac competent *E. coli*, and the recombinant pFastBac vectors were extracted from competent *E. coli*. The transfection of vectors was performed using Cellfectin II with SF9 cells following the manufacturer’s recommended procedure (Invitrogen, Carlsbad, CA, USA), and Sf9 cells were cultured in serum-free SF900 III medium.

### 2.7. The Expression and Culture of Virus-Like Particles 

Sf9 cells were infected with rBV vectors expressing the *T. gondii* SAG1 and influenza matrix protein 1, and VLPs were expressed in Sf9 cells. Briefly, the culture supernatant was harvested at 5 days after infection and centrifuged at 5000 rpm for 20 min at 4 °C, and the cellular debris was carefully removed. The VLPs in supernatant were filtered through a 0.45 μm membrane filter system, and the VLPs were pelleted at 30,000 rpm by high-speed centrifugation for 1 h at 4 °C. The VLPs were further purified through a 10%-20%-30%-60%-80% discontinuous sucrose gradient at 27,000 rpm for 1 h at 4 °C. The VLP layers or bands were carefully collected between 30% and 80%. The VLPs were pelleted at 30,000 rpm for 1 h at 4 °C and resuspended in 0.5X PBS at 4 °C overnight. The VLP concentration was quantified with an Eppendorf BioSpectrometer (Eppendorf, Seoul, Korea), which was stored at 4 °C until use. 

### 2.8. The Evaluation of VLPs through Western Blot Analysis 

*T. gondii* SAG1 or influenza matrix protein 1, and the VLPs containing the *T. gondii* SAG1 and influenza matrix protein 1 were confirmed through the western blot method. The *T. gondii* SAG1 and influenza matrix protein 1 were probed with the polyclonal mouse anti-*T. gondii* antibody (prepared from the sera of *T. gondii*-infected mice) and the monoclonal mouse anti-influenza A matrix protein 1 antibody (MyBioSource, Inc., San Diego, CA, USA, and Invitrogen, Carlsbad, CA, USA), respectively. Subsequently, after washing, they were confirmed after incubation with a secondary antibody (goat anti-mouse IgG poly-HRP antibody, Invitrogen, Carlsbad, CA, USA) at room temperature for 1 h.

### 2.9. VLP Immunization and Parasite Infection 

**Figure 1:**
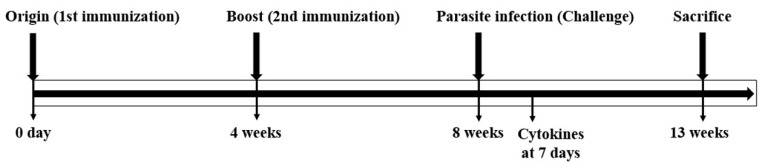
The experimental schedule for vaccination and *Toxoplasma gondii* infection. The mice were immunized twice with virus-like particles (VLPs) at origin (0 day) and boost (4 weeks), respectively. The parasite infection (challenge) was performed at 4 weeks after the second immunization.

The Balb/c mice (5–6 weeks/female) were divided into groups (*n* = 10/group) as follows: (1) the immunization step: the normal group and the immunized group, and (2) challenge step: the normal group, the non-immunized group infected with the parasite, and the immunized parasite infection group. The mice were immunized twice through intramuscular administration with SAG1-VLPs (120 μg) at 4 week intervals. The sera were collected from mice at 4 weeks after the first and second immunization, respectively. In addition, the mice were infected with *T. gondii* RH (8 × 10^4^ tachyzoites) through oral administration at 4 weeks after the second immunization, and the sera were collected at 7 days after parasite infection. The mice were carefully observed daily, and food and water were provided ad libitum to all the mice during the experimental period. To evaluate the effect of the vaccination regarding SAG1-VLPs, the survival rates of all the groups were carefully observed daily after the immunization and parasite infection. The experimental schedule for vaccination and parasite infection (challenge) is shown in Figure 1. 

### 2.10. The Evaluation of Antibody Responses in Sera 

Blood samples were collected at 4 weeks after the first and second immunization, and at 7 days after parasite challenge, respectively. The sera were used to detect the antibody response produced after the immunization and *T. gondii* infection. To evaluate the *T. gondii*-specific antibody response, Horseradish peroxidase (HRP)-conjugated goat anti-mouse immunoglobulin G (IgG), G1 (IgG1), G2a (IgG2a), and A (IgA) were purchased from R&D Systems Inc., (Minneapolis, MN, USA) and Southern Biotech (Birmingham, AL, USA). The IgG, IgG1, IgG2a, and IgA antibody responses were evaluated by the enzyme-linked immunosorbent assay (ELISA). Briefly, a 96-well plate was coated with 100 μL of *T. gondii* antigen (4 μg/mL)/well at 4 °C overnight. After removing the antigen solution, the plate was incubated with blocking buffer containing 0.1% BSA for 1 h. The sera were diluted 1:50 with 0.5X PBS, and 100 μL of sera was added into each well of the plate in duplicate. The plate was incubated for 1 h at 37 °C and washed with 0.5X PBS containing 0.02% Tween 20. Subsequently, the plate was incubated with an HRP-conjugated secondary antibody diluted 1:1000 in 0.5X PBS for 1 h at 37 °C. After washing with 0.5X PBST, the sera were measured using an ELISA reader following the manufacturer’s recommended procedure. All the samples were stored at −80 °C until use.

### 2.11. Cytokine Analysis in the Spleen 

To confirm the production of cytokines in the spleen through *T. gondii* infection, the spleen of mice was collected from all of the groups at 7 days after parasite challenge. The groups were divided into three groups (*n* = 10/group) as follows: the normal group, the non-immunized group infected with the parasite (non-immunized infection group), and the parasite-infected group immunized with SAG1-VLPs (immunized infection group). Briefly, after the spleen was disrupted through cutting and tissue homogenizer at 4 °C, the mixture was separated by centrifugation at 12,000 rpm for 5 min at 4 °C. The cellular debris was removed, and the supernatants were carefully collected. The cytokines (interleukin (IL): IL-1β, IL-4, IL-6, IL-12, tumor necrosis factor alpha (TNF-α), and Interferon (IFN)-γ) were respectively measured in the normal group, the non-immunized infection group, and the immunized infection group, according to the manufacturer’s instructions through cytokine ELISA kits (ThermoFisher Scientific, San Diego, CA, USA) and multi-cytokine kits (Qiagen, Hilden, Germany). The samples were stored at −80 °C until use. 

### 2.12. Evaluation of the Proliferation of T. gondii through Immunized Sera

To evaluate the inhibitory effect of the proliferation of *T. gondii* through specific sera immunized with SAG1-VLPs, the sera were collected from mice at 4 weeks after boost immunization, and 150 μL of the sera was incubated with *T. gondii* RH (1500 tachyzoites). The mixture was incubated at 37 °C for 6 h, mixed once with 1 h interval, and injected into mice through the intraperitoneal (IP) route. The mixture of *T. gondii* RH and non-immunized sera was also injected into mice, which was used as the control group. The groups were carefully observed daily during the experimental period. The groups were divided into two groups (*n* = 10/group) as follows: the non-immunized group infected with the parasite (control group) and the parasite infection group immunized with SAG1-VLPs (the immunized group). The parasites were collected from the abdominal cavity of mice at 7 days after infection. The survival rates of the parasites were counted with a hemocytometer chamber through trypan blue under a microscope. 

### 2.13. The Viability of Splenocytes after Immunization

To evaluate the cell viability of splenocytes after the second immunization, the spleen of mice was collected from all groups at 4 weeks after boost immunization. The cell groups were divided into three groups as follows: the normal group, the non-immunized group infected with TLA (non-immunized infection group), and the TLA infection group immunized with SAG1-VLPs (immunized infection group). Briefly, splenocytes were seeded in a 48-well plate (3 × 10^5^/well), and the cells were cultured in RPMI medium 1640 containing 2 mM l-glutamine, supplemented with 15% decomplemented fetal bovine serum (FBS) in a humidified atmosphere containing 5% CO_2_ in air at 37 °C. The cells were incubated with or without TLA (1 μg/mL)/well for 72 h at 37 °C, and their viabilities were then measured by the MTT assay. 

### 2.14. Survival Rate of Mice through T. gondii Infection 

To evaluate the protective effect and vaccine efficacy of SAG1-VLPs against *T. gondii* infection, the mice were infected with *T. gondii* RH (8 × 10^4^ tachyzoites) through oral administration at 4 weeks after boost immunization. The groups were divided into three groups (*n* = 24/group) as follows: the normal group, the non-immunized group infected with the parasite (non-immunized infection group), and the parasite-infected group immunized with SAG1-VLPs (immunized infection group). The survival rates of all groups were carefully observed daily after parasite infection during the experiment. 

### 2.15. Statistical Analysis 

All results were expressed as mean ± standard deviation (S.D.). Statistical analysis of the data was performed using Student’s *t*-test and analysis of variance (ANOVA). A value of * *p* < 0.05 was considered to be significant.

## 3. Results 

### 3.1. Production and Expression of T. gondii-Specific SAG1-VLPs 

**Figure 2:**
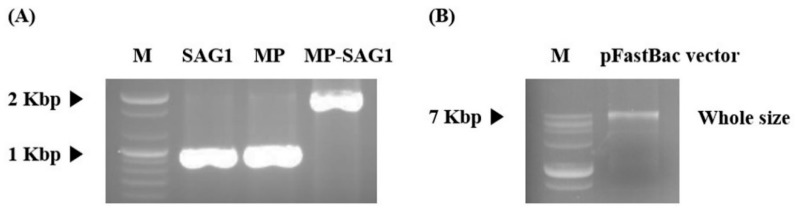
The analysis of specific genes on VLPs containing *T. gondii* surface antigen 1 (SAG1) and influenza matrix genes. (**A**) *T. gondii* SAG1 gene (1011 bp), influenza A matrix protein 1 gene (MP; 1027 bp), and SAG1-matrix protein 1 gene (2038 bp) cloned into the pFastBac vector. (**B**) The pFastBac vector containing *T. gondii* SAG1 and matrix protein 1 genes (whole size; 6813 bp). M: marker; MP: matrix protein 1 gene; MP-SAG1: influenza A matrix protein 1 and SAG1 genes cloned into the vector.

**Figure 3:**
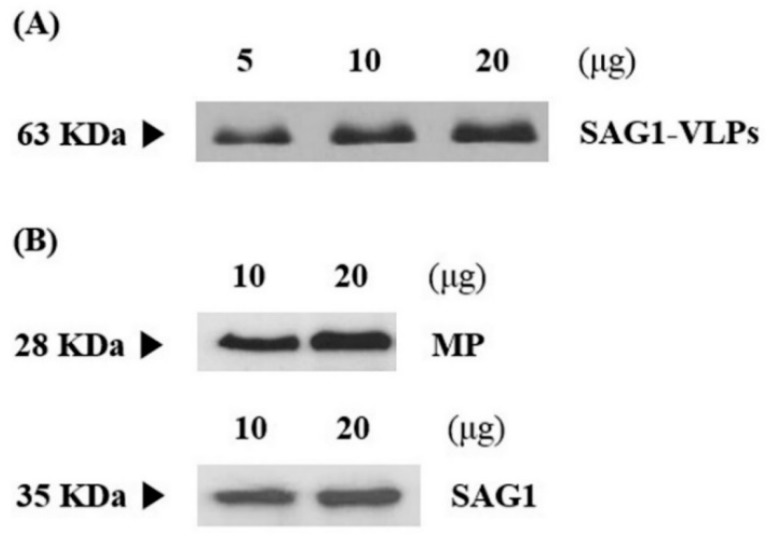
Western blot analysis of specific factors that construct VLPs. (**A**) SAG1-VLPs (63 KDa) expressed from the pFastBac vector containing SAG1 and matrix protein 1. (**B**) The matrix protein 1 (MP; 28 KDa) expressed from the pFastBac vector without *T. gondii* SAG1, and the SAG1 (35 KDa) expressed from the pFastBac vector without matrix protein 1. The proteins were loaded 5, 10, and 20 μg on SDS-PAGE, respectively. The polyclonal mouse anti-*T. gondii* antibody and monoclonal mouse anti-matrix protein 1 antibody were used as a probe to detect SAG1 and matrix protein 1, respectively.

**Figure 4:**
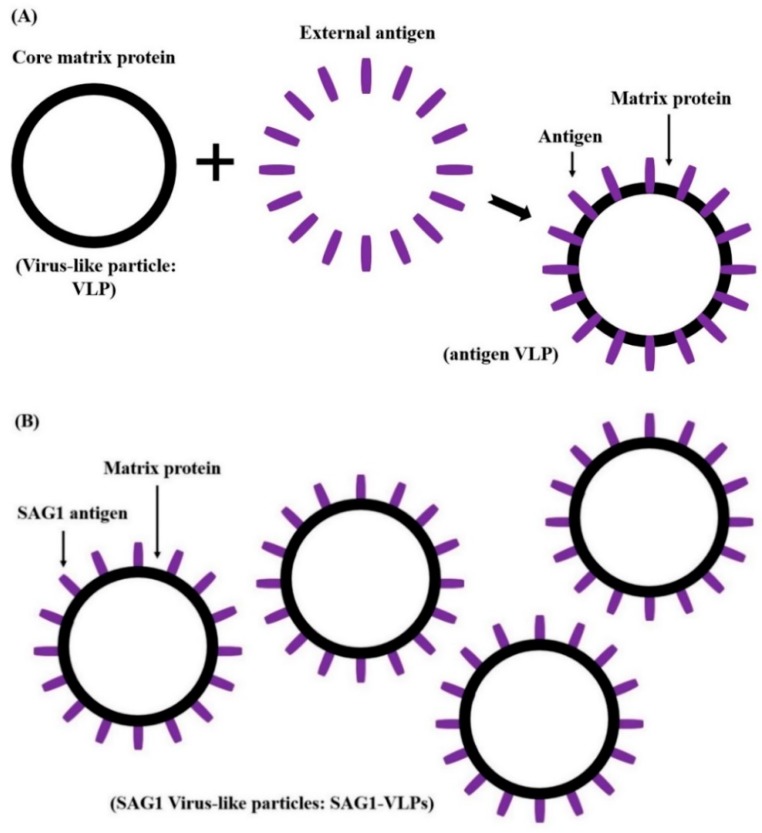
The composition and diagram of antigen VLPs. The VLP consists of a matrix protein and external antigen, which are integral to the form and generation of the VLP. (**A**) The component factors of antigen-VLP, and (**B**) the structure of SAG1-VLPs containing the SAG1 antigen.

*T. gondii* consists of a specific network and structure, including various and unique micro-organelles. This study developed a vaccine candidate that induces effective immunogenicity and vaccination efficacy against *T. gondii* infection using the influenza matrix and SAG1 among various subcellular organelles of the parasite. *T. gondii*-specific SAG1 and influenza matrix genes were amplified by RT-PCR and PCR with their specific primers using the total RNA of *T. gondii* and the synthesized matrix genes respectively, and their specific genes were cloned into a pFastBac vector (Figure 2). Subsequently, *T. gondii*-specific SAG1-VLPs containing *T. gondii* SAG1 and influenza matrix protein were generated in Sf9 cells, and the VLPs were confirmed through western blot analysis (Figure 3). The generation principle of VLPs containing *T. gondii* SAG1 and influenza matrix protein is shown in Figure 4. Recently, various studies regarding vaccination have been reported, especially research on vaccines against parasites. However, recent studies showed the limitation that they could not inhibit toxoplasmosis induced by *T. gondii* infection. In this regard, the results of this study provide the accurate information on VLPs through their molecular weight when matrix protein 1 includes or does not include the specific antigen as spikes, which indicates the potential and utility of them as an antigen delivery system of vaccination for preventing *T. gondii* infection. Therefore, these results clearly indicate the SAG1-VLPs formed through the *T. gondii* SAG1 antigen and the matrix protein 1, as well as the VLP formed through only matrix protein 1, without the specific antigen or spikes. 

### 3.2. Evaluation of the Antibody Response through SAG1-VLP Immunization

**Figure 5:**
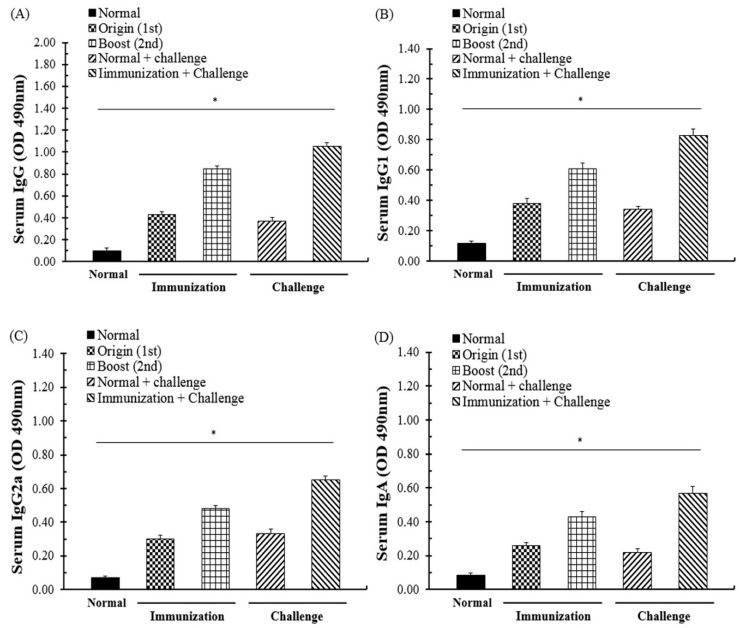
*T. gondii* antibody response after immunization and *T. gondii* infection. The sera of mice were collected at 4 weeks after the first and second immunization, and at 1 week after *T. gondii* infection, respectively. (**A**) IgG, (**B**) IgG1, (**C**) IgG2a, and (**D**) IgA antibody responses were evaluated using the sera at the time point, respectively. The groups were divided as follows: (1) the immunization step: the normal group and immunization group (the group immunized with SAG1-VLP), and (2) the parasite infection step: the normal group, normal (non-immunized) group infected with *T. gondii*, and *T. gondii* infection group immunized with SAG1-VLPs.

The effects of antibody generation and the immunogenicity of SAG1-VLPs against *T. gondii* were evaluated through the sera of mice at 4 weeks after the first and second immunization, respectively, and the groups were carefully observed daily after the immunization during the experiment. After the first immunization, the antibody (IgG, IgG1, IgG2a, and IgA) levels of mice were significantly increased in all of the groups, and in particular, the IgG level was strongly increased compared to other antibody groups (Figure 5). Furthermore, after the second immunization, the antibody levels were increased in all of the groups, and the generation rates of IgG and IgG1 were higher than those of other groups. The results indicate that *T. gondii*-specific antibodies were effectively induced in the body by being strongly produced through the interaction between memory B cells and immune cells after the first and second immunization. These results demonstrate that SAG1-VLP immunization not only effectively induces the immunogenicity through the *T. gondii*-specific antigen, but also continuously increases the production of specific antibodies after the immunization. 

### 3.3. Specific Antibody Response through Immunization and T. gondii Infection 

**Figure 6:**
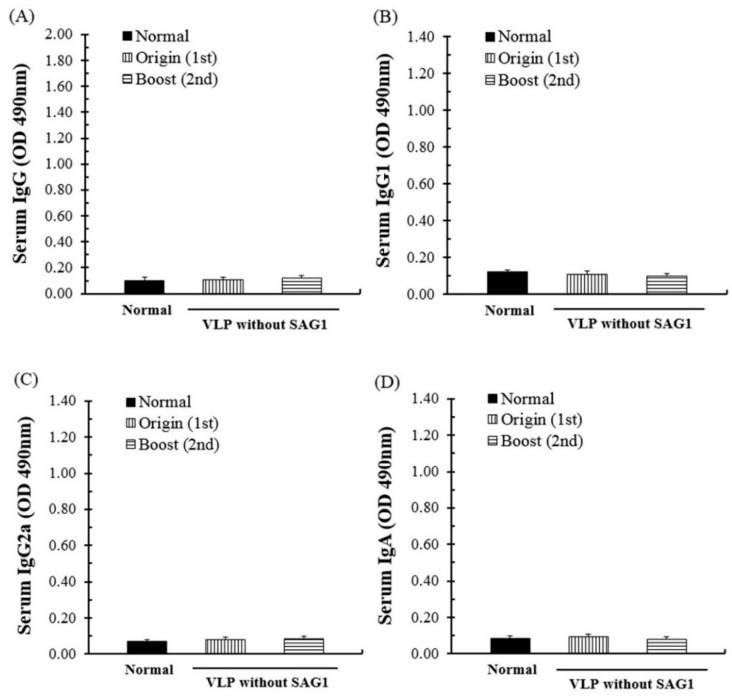
*T. gondii* antibody response after the immunization of VLPs alone without the SAG1 antigen. The sera of mice were collected at 4 weeks after the first and second immunization, respectively. (**A**) IgG, (**B**) IgG1, (**C**) IgG2a, and (**D**) IgA antibody responses were evaluated using the sera at the time point, respectively. The groups were divided as follows: the immunization step: the normal group and immunization group (the group immunized with VLP alone, without SAG1).

The specific antibody response and its generative effects regarding SAG1-VLP immunization were measured using the sera of mice at 7 days after parasite challenge. The IgG, IgG1, IgG2a, and IgA antibody responses were increased more than those of the boost immunization in all groups, and the antibody levels were increased gradually in the immunized group (Figure 5). The results indicate that the specific antibodies effectively induced a systemic reaction against the parasite by being rapidly produced in the body after parasite infection. In addition, although mice were immunized with VLPs alone without the SAG1 antigen, they had no specific antibody responses compared with the normal group, which indicates the potential of them as a specific antigen delivery system for preventing *T. gondii* infection (Figure 6). These results show that SAG1-VLPs not only increase the protective actions and immunogenicity against *T. gondii* infection by effectively inducing the production of specific antibodies through the first and second immunization, but also induce or accelerate the progressive maturation of antibodies generated through the immune system. 

### 3.4. The Change of Cytokines of Immune Cells after T. gondii Infection 

**Figure 7:**
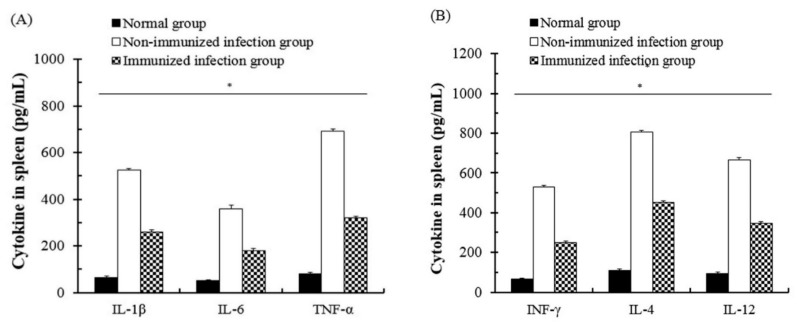
The change of production of cytokines expressed from the spleen. The spleens were collected from mice at 7 days after *T. gondii* infection (challenge), which was used for detecting the expression of different cytokines (interleukin (IL)-1β, IL-4, IL-6, IL-12, TNF-α, and INF-γ). (**A**) IL-1β, IL-6, and TNF-α. (**B**) INF-γ, IL-4, and IL-12. The groups were divided into three groups as follows: the normal group, the non-immunized group infected with *T. gondii* (the non-immunized infection group), and the *T. gondii* infection group immunized with SAG1-VLPs (the immunized infection group).

The change and interaction of cytokines through SAG1-VLP immunization were measured using the spleen of mice, and the spleen was collected from all groups at 7 days after *T. gondii* infection. The groups were carefully observed daily during the experiment. As shown in Figure 7, the cytokines such as IL-1β, IL-4, IL-6, IL-12, TNF-α, and IFN-γ were measured in all groups respectively, and the levels of IL-1β, IL-4, and TNF-α were strongly increased in the non-immunized group compared to the immunized group. In particular, the levels of cytokines in the immunized groups were markedly reduced compared to the non-immunized group. In addition, there were no significant changes of cytokines in normal groups. The results indicate that the levels of cytokines gradually decreased in the body because SAG1-VLPs induced a decrease of stimulation and the systemic reaction caused by the parasite infection by continuously inducing the generation of *T. gondii-*specific antibodies through twice immunizations. These results clearly show that SAG1-VLP immunization not only decreases the production of cytokines (IL-4, IL-12, and IFN-γ) associated with the infection of pathogens in the host, but also effectively inhibits the inflammatory cytokines (IL-1β, IL-6, and TNF-α) after *T. gondii* infection. 

### 3.5. The Neutralizing Effect of the Immunized Sera against T. gondii 

**Figure 8:**
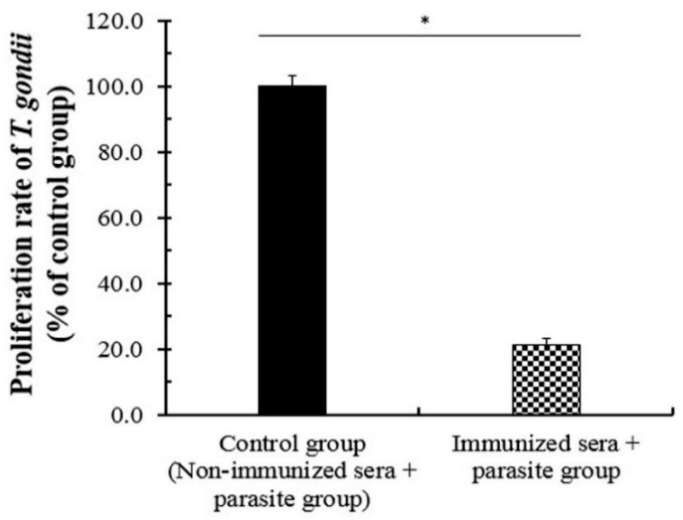
The inhibitory effect of the immunized sera against the proliferation/growth of *T. gondii*. The sera of mice were collected at 4 weeks after boost immunization. The sera were mixed with *T. gondii*, which was injected into the abdominal cavity of normal mice. The parasites were collected from the abdominal cavity at 7 days after intraperitoneal (IP) challenge, which was counted. The groups were divided into two groups as follows: the non-immunized sera group infected with *T. gondii* (control group), and the immunized sera group infected with *T. gondii* (immunization group).

The neutralizing effects of the immunized antibodies against pathogens are known as an important function and a major factor of the vaccination effect [28,29]. For this reason, the protective effects of the specific antibody generated by *T. gondii*-specific SAG1-VLPs were evaluated using the immunized sera at 4 weeks after boost immunization. The mixture of *T. gondii* and immunized sera or the non-immunized sera was injected into the abdominal cavity of mice, respectively, which was measured at 7 days after *T. gondii* infection. The proliferation of *T. gondii* was markedly inhibited in the immunization group compared to the non-immunized group, and their proliferation rates were lower than 25% (Figure 8). The results show that SAG1-VLPs consistently induce the generation of *T. gondii*-specific antibodies as a specific antigen or immunogenicity against *T. gondii*. These results demonstrate that SAG1-VLP immunization not only inhibits the survival of *T. gondii* by effectively inducing the progressive maturation and activation of *T. gondii*-specific antibodies, but also neutralizes or strongly decreases the proliferation/growth of *T. gondii* through the specific antibodies.

### 3.6. Cell Viability of Splenocytes after the Immunization of SAG1-VLPs 

**Figure 9:**
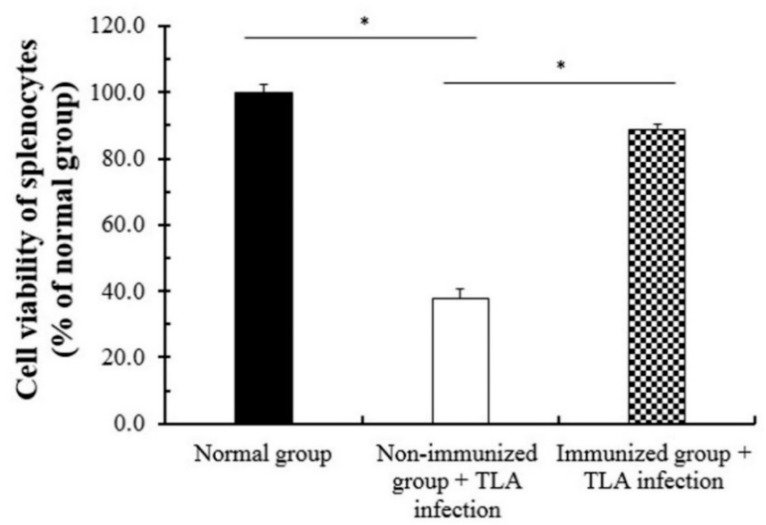
The viability of splenocytes after immunization. The splenocytes were collected from the spleen of mice at 4 weeks after boost immunization, and the cells were incubated with *T. gondii* lysate antigen (TLA) (1 μg/mL) or without TLA for 72 h. The cell viability was measured by the MTT assay. The groups were divided into three groups as follows: the normal group, the non-immunized group infected with TLA (the non-immunized TLA infection group), and the TLA-infected group immunized with SAG1-VLPs (the immunized TLA infection group).

To confirm the protective effect of the *T. gondii*-specific antibody through SAG1-VLP immunization, the cell viability of splenocytes was measured using splenocytes collected from mice at 4 weeks after boost immunization. The cell groups were divided into three groups: the normal group, the non-immunized TLA infection group, and the immunized TLA infection group. After TLA infection, the viability of spleen cells was markedly decreased in the non-immunized TLA infection group compared with the immunized TLA infection group, which was confirmed through the differences in the survival rates (Figure 9). In particular, the cell viability of the immunized TLA infection group was higher than 83%, whereas the viability of the non-immunized group was lower than 40%. The results indicate that SAG1-VLPs effectively induce the selective protective action and/or activity against TLA through specific antibodies generated by twice immunizations, but also increase the viability of spleen cells through the activity of *T. gondii-*specific antibodies. These results show that SAG1-VLP immunization may prevent toxoplasmosis which is induced by parasite infection by strongly inhibiting or blocking the survival of *T. gondii* through the production of specific antibodies. 

### 3.7. Evaluation of the Survival Rate of Immunized Mice after T. gondii Infection 

**Figure 10:**
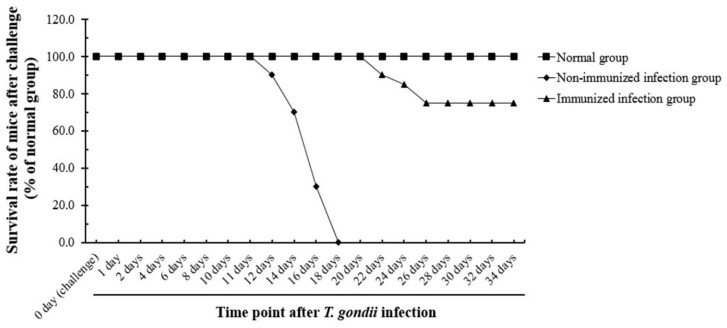
The survival rate of mice after *T. gondii* infection. The groups were observed daily for 35 days after *T. gondii* infection (challenge). The non-immunized group all died within 18 days after the parasite infection, whereas the group immunized with SAG1-VLPs showed a survival rate of 75%. The groups were divided into three groups as follows: the normal group, the non-immunized group infected with *T. gondii* (the non-immunized infection group), and the *T. gondii* infection group immunized with SAG1-VLPs (the immunized infection group).

The vaccine efficacy and protective effects of SAG1-VLP immunization after parasite infection were evaluated through the mice infected by *T. gondii* at 4 weeks after boost immunization. The groups were divided into the normal group, the non-immunized infection group, and the immunized infection group, and the mice were carefully observed in all groups daily for 35 days after parasite infection. The survival rates of the immunized infection group were significantly increased compared to the non-immunized infection group, and their survival rates were 75% for 35 days. However, the non-immunized infection group all died within 18 days after infection (Figure 10). The results show that SAG1-VLPs not only increase the survival of mice by effectively inhibiting the proliferation of *T. gondii* through the *T. gondii*-specific antibody generated after immunization, but also cause the selective inhibitory effect/action against the parasite through the protective effect and vaccine efficacy against *T. gondii* infection. These results demonstrate that *T. gondii*-specific SAG1-VLP can be effectively used as a potential vaccine candidate for preventing or inhibiting toxoplasmosis. 

## 4. Discussion 

For the past decades, in spite of constant efforts to improve public health in various fields worldwide, the infectious diseases, including Ebola, middle east respiratory syndrome (MERS), severe acute respiratory syndrome (SARS), Zika, influenza, tuberculosis, malaria, amoebiasis, and leishmaniasis, have consistently induced a global public health concern owing to their potential to become pandemics. Infectious pathogens are still increasing and evolving their adaptability to environmental variation through infectivity to the host and their viability. Furthermore, many people are still dying by infectious diseases such as influenza, tuberculosis, dengue fever, and malaria every year worldwide. In this aspect, the international organizations and companies, including WHO and global pharmaceuticals, are persistently supporting various studies on treating and preventing acute or chronic infectious diseases, which are being focused on studies for developing vaccines and novel drugs against pathogens. Recently, animal infectious diseases, such as Avian influenza (AI) and African swine fever (ASF), are causing a pandemic worldwide, and they have the potential to be transmitted to humans by overcoming limitations of the interspecies barrier though their mutation. Furthermore, although studies for preventing or inhibiting parasitosis and zoonosis have been performed in various fields worldwide, effective drugs have not yet been launched clinically, which is consistently causing a public health concern worldwide. Recently, the number of people living with companion animals (cats and dogs, etc.) has been increasing globally, but infectious diseases which are induced by them have been overlooked and have not attracted attention. 

For these reasons, this study focused on toxoplasmosis among zoonosis that is transmitted by various infectious pathways. Toxoplasmosis is a zoonotic parasitosis that infects humans through feces of cats and dogs, including vertebrates. In particular, *T. gondii* induces not only serious complications in pregnant women, immune deficient patients, and HIV patients, but also cerebral calcification and toxoplasmic encephalitis through brain infection. *T. gondii* infection regulates the response of *T. gondii* IgG seroprevalence that specifically responds to *T. gondii* in patients. *T. gondii* may affect the production and interaction of cytokines, as well as induce an imbalance of the immune response through a change of the cytokines after infection, which results in immune deficiency by weakening homeostasis of the immune system in the host [30,31,32,33]. Moreover, it is known that *T. gondii* blocks or inhibits the signal pathways of cell cycle initiators or the apoptotic stage in host cells. *T. gondii* promotes its proliferation by rapidly forming PVM or regulating cell cycle factors of the host for survival after invasion, which finally increases the viability of *T. gondii* in the host [34,35]. Recently the subcellular organelles of *T. gondii*, such as dense granules (GRAs), the rhoptry, the microneme, and the inner membrane complex, have been used as major recombinant proteins and/or antigens for vaccine candidates, as well as major targets for inhibiting *T. gondii* infection. Furthermore, various studies regarding vaccine development have been reported, particularly vaccine researches against *T. gondii* infection [36,37,38,39,40], as well as the surface antigens and recombinant chimeric proteins of *T. gondii* [41,42,43,44]. Nevertheless, the recent studies have still difficulties in relation to parasite infection and its treatment. 

From these perspectives, this study tried to find feasible alternatives for overcoming toxoplasmosis induced by *T. gondii* infection, and to discover an effective vaccine candidate for preventing and inhibiting the parasite infection and zoonosis. In particular, the various components and factors of both the virus and *T. gondii* can be effectively utilized or applied in vaccine development fields. For this reason, this study focused on developing a novel vaccine candidate through the *T. gondii* antigen and VLP of the virus as one of the various strategies for developing novel vaccines against parasite infection, and evaluated the protective effect and efficacy of a novel vaccine candidate against *T. gondii* infection. In the present study, *T. gondii*-specific SAG1 and influenza matrix genes were cloned into a pFastBac vector, which was clearly confirmed through RT-PCR and PCR analysis. The expression of *T. gondii* SAG1-VLPs was confirmed by western blot analysis. The results clearly indicated the SAG1-VLPs expressed by *T. gondii*-specific SAG1 antigen and influenza matrix protein 1 as well as the VLPs without the antigen, which remedied the uncertainty and incorrect results of recent VLP studies by clearly presenting the difference regarding the formation of VLP. In addition, the antibody (IgG, IgG1, IgG2a, and IgA) levels of mice were significantly increased in all groups after origin and boost immunization, and the antibodies were effectively produced in the group immunized with SAG1-VLPs compared to non-immunized groups. These results show that SAG1-VLPs not only effectively caused the immunogenicity by inducing the production of specific antibodies through the parasite-specific antigen after twice immunizations, but also persistently increased the protective actions against *T. gondii* infection by effectively inducing or accelerating the progressive maturation of specific antibodies. Although the cytokines (IL-1β, IL-4, IL-6, IL-12, TNF-α, and IFN-γ) were increased in all groups after the parasite infection, the cytokine levels of the group immunized with SAG1-VLPs were markedly reduced compared to the non-immunized infection group. The results clearly indicated that SAG1-VLP immunization significantly reduced the production of cytokines which are induced by the parasite infection by effectively generating *T. gondii*-specific antibodies through twice immunizations. In particular, the neutralizing effect of the immunized antibodies is an indicator which shows the inhibition and preventive ability against pathogens as a major factor of the vaccination effect. The survival rates of *T. gondii* were significantly inhibited in the group immunized with SAG1-VLPs compared to the non-immunized group, and the cell viability of TLA-treated splenocytes was markedly decreased in the non-immunized group. Furthermore, the survival rate of the group immunized with SAG1-VLPs was 75% for 35 days after *T. gondii* infection, whereas the non-immunized infection group all died within 18 days after the parasite infection. The results demonstrate that SAG1-VLPs effectively induced the activity of antibodies as well as the vaccination effect/action against *T. gondii* infection through immunization. 

Taken together, this study showed the anti-parasitic vaccination effect/ability of SAG1-VLPs against *T. gondii* infection, and developed an effective and novel antigen delivery system against *T. gondii* using the influenza matrix protein and *T. gondii*-specific SAG1, which was clearly demonstrated through twice immunizations and parasite infection. The SAG1-VLPs showed the effective immunogenicity action/function by consistently inducing or promoting the production of the *T. gondii-*specific antibody after twice immunizations. The results indicated that SAG1-VLP immunization effectively decreased the inflammatory cytokines (IL-1β, IL-6, and TNF-α), as well as the anti-inflammatory cytokines (IL-4, IL-12, and IFN-γ) after *T. gondii* infection. Furthermore, the sera immunized with SAG1-VLPs showed a neutralizing activity/effect by inhibiting the proliferation and growth of *T. gondii* through specific antibodies generated by the immunization, and the immunization of SAG1-VLPs exhibited a protective effect against TLA by effectively increasing and activating the viability of splenocytes infected with TLA. In particular, SAG1-VLP immunization effectively increased the survival rate of the mice by specifically inhibiting or blocking the death of mice which is induced by the proliferation of *T. gondii* after *T. gondii* infection. In addition, the vaccination effects of SAG1-VLPs were clearly confirmed through both in vitro and in vivo experiments in this study. 

## 5. Conclusions

The results of this study show that *T. gondii-*specific SAG1-VLPs effectively induce an immunogenicity function as a specific antigen delivery system for preventing toxoplasmosis and *T. gondii* infection, as well as vaccine efficacy against *T. gondii*. In particular, the results indicate that SAG1-VLPs not only induce the anti-*T. gondii* activity through the neutralizing effect of *T. gondii-*specific antibodies, but also increase the progressive maturation and activation of antibodies generated by the interaction between memory B cells and immune cells after immunization. The results of this study demonstrate that SAG1-VLPs effectively cause the selective *T. gondii* vaccination effect and/or anti-*T. gondii* activity by effectively inhibiting or blocking the survival and proliferation of *T. gondii* through the specific antibodies generated after immunization. In addition, these results demonstrate that SAG1-VLP could be utilized or applied as an efficient antigen delivery system for preventing or inhibiting other infectious diseases. Therefore, this study indicates clearly that SAG1-VLP has the potential to be effectively used as a promising *T. gondii* vaccine candidate for developing novel anti-*T. gondii* vaccines through preclinical studies in the near future.
